# Shock loss measurements in non-ideal supersonic flows of organic vapors

**DOI:** 10.1007/s00348-022-03465-y

**Published:** 2022-07-11

**Authors:** Camilla C. Conti, Alberto Fusetti, Andrea Spinelli, Alberto Guardone

**Affiliations:** 1 Department of Aerospace Science and Technology, Politecnico di Milano, Via La Masa 34, 20156 Milano, Italy; 2 Department of Energy, Politecnico di Milano, Via Lambruschini 4a, 20156 Milano, Italy

## Abstract

This paper presents the first ever direct measurements of total pressure losses across shocks in supersonic flows of organic vapors in non-ideal conditions, so in the thermodynamic region close to the liquid–vapor saturation curve and the critical point where the ideal gas law is not applicable. Experiments were carried out with fluid siloxane MM (hexamethyldisiloxane, C$_6$H$_{18}$OSi$_2$), commonly employed in medium-/high-temperature organic Rankine cycles (ORCs), in the *Test Rig for Organic VApors* (TROVA), a blowdown wind tunnel at the Laboratory of Compressible fluid dynamics for Renewable Energy Applications (CREA lab) of Politecnico di Milano. A total pressure probe was inserted in superheated MM vapor flow at Mach number $\sim 1.5$ with total conditions in the range $215-230~^\circ \hbox {C}$ and $2-12~\hbox {bar}$ at varying levels of non-ideality, with a compressibility factor evaluated at total conditions between $Z_\text {T}=0.68-0.98$. These operating conditions are representative of the first-stage stator of ORC turbines. Measured shock losses were compared with those calculated from pre-shock quantities by solving conservation equations across a normal shock, with differences always below $2 \%$ attesting a satisfactory reliability of the implemented experimental procedure. An in-depth analysis was then carried out, highlighting the direct effects of non-ideality on shock intensity. Even at the mildly non-ideal conditions with $Z_\text {T}\gtrsim 0.70$ considered here, non-ideality was responsible for a significantly stronger shock compared to the ideal gas limit at same pre-shock Mach number, with differences as large as $6\%$.

**Graphical abstract:**

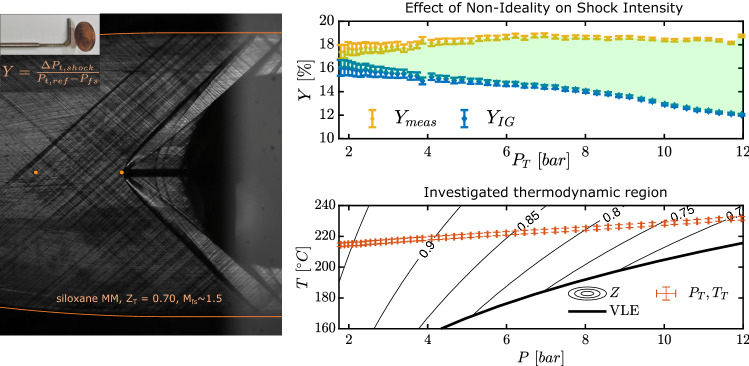

## Introduction

Non-ideal compressible fluid dynamics (NICFD) is a branch of gas dynamics that studies flows of dense vapors occurring in the close proximity of the vapor–liquid equilibrium and the critical point, so in conditions in which the ideal gas law does not properly describe the thermodynamics involved (Thompson 1971). *Compressibility factor* *Z* is defined in Eq. (1) where *P* is pressure, *T* is temperature, $\rho$ is density and *R* is the gas constant.

1 $$\begin{aligned} Z = \dfrac{P}{R T \rho } \end{aligned}$$

It represents the departure of the fluid volumetric behavior from that of an ideal gas. It is therefore identically equal to 1 in case of ideal gas behavior and possibly differs from 1 otherwise: It is thus an indication of the level of non-ideality. The term *non-ideal* in NICFD indeed refers to this latter aspect, which has a number of direct gas dynamic “side effects” distinctly separating such flows from those of ideal gases. For example, isentropic expansions show a non-ideal dependence on total conditions, analogously to shock waves, which no longer depend on the pre-shock Mach number only.

Non-ideal flows occur in a vast range of engineering processes, from rocket propulsion to industrial and chemical activities. Examples are the oil and gas, heat pumps and refrigeration fields, and even pharmaceuticals production with the use of the rapid expansion of supercritical solutions (Helfgen et al. 2003). In the power generation field, non-ideal flows occur in supercritical carbon dioxide ($\text {sCO}_2$) power cycles and are particularly important in organic Rankine cycles (ORCs), one of the most relevant application fields for NICFD. ORCs are preferred to conventional steam cycles when low to medium source temperature and low to medium power output are considered, thanks to their low cost, plant simplicity and thermodynamic efficiency. Fluids usually employed in ORCs feature high complexity and molecular weight, and turbine expansion occurs in the dense gas region near the saturation curve and the critical point. As a result, turbine flows are highly supersonic and show strong non-ideal flow effects (Romei et al. 2020), requiring accurate design tools accounting for these aspects in order to achieve high turbine efficiency, which in turn strongly impacts cycle efficiency (Colonna et al. 2015; Macchi and Astolfi 2016).

Comparison of numerical tools for design and analysis, ranging from preliminary loss correlations to full computational fluid dynamics (CFD) simulations, with experiment is relatively rare. This is because detailed experimental data characterizing non-ideal flows for ORC applications are currently not widely available in the open literature due to the intrinsic difficulties in running dedicated experimental facilities. ORC working fluids, like many others of interest in the NICFD field, are liquids at standard room temperature and pressure. Typical ORC inlet turbine flows are instead at saturated, superheated or supercritical conditions, with temperatures and pressures  in the range 100–$400~^\circ \hbox {C}$ and 10–$50~\text {bar}$ (Macchi and Astolfi 2016). Thus, in order to reproduce realistic conditions in a wind tunnel, a closed gas cycle or a phase transition thermodynamic cycle must be put in place (Spinelli et al. 2020). These are noticeably more complicated and expensive with respect to operation with incondensable gases such as air, where compressed air storage tanks or continuous loops are often sufficient to carry out an experimental campaign. Moreover, measurement procedures are also more complex due to the high fluid temperature involved and condensation issues in pneumatic lines. Also, the non-ideal flow field dependence on stagnation conditions significantly increases the number of flows that have to be experimentally reproduced for a complete characterization. Indeed, a polytropic ideal gas flow in a fixed geometry features trends in Mach number, pressure and temperature ratios that are independent of the actual total conditions and only depend on molecular complexity through the specific heat ratio $\gamma$. In case of non-ideal flows instead, testing needs to cover the whole range of possible operating total temperature and pressure conditions.

Despite these difficulties, several active plants exist and are starting to provide valuable experimental data, mostly on relatively simple yet extremely useful geometries such as converging–diverging nozzles. These allow to reproduce elementary flows important for fundamental NICFD studies and are also the simplest geometry representative of blade passages in ORC turbines. Among these, so-called *nozzle-fitted facilities* is the *Test Rig for Organic VApors* (TROVA) (Spinelli et al. 2013) at the Laboratory of Compressible fluid dynamics for Renewable Energy Applications (CREA Lab) of Politecnico di Milano, where all the experimental campaigns concerning the present work were carried out. Other plants of this kind are the ORCHID (Head et al. 2016) at TU Delft, the CLOWT (Reinker et al. 2017) at Muenster University of Applied Sciences and the dense gas blowdown facility at Imperial College London (Robertson et al. 2020).

Several turbine-fitted facilities instead including all typical components of an organic Rankine cycle also exist, such as the *LUT micro-ORC test rig* at Lappeenranta - Lahti University of Technology (Turunen-Saaresti et al. 2017). The ORCHID at TU Delft is designed to also operate with a turbine instead of a nozzle, but testing to date was performed with the latter only. These test rigs are mainly devoted to performance measurement of the different components and of the overall thermodynamic cycle and are less suited to provide detailed flow information than nozzle-fitted facilities.

Due to the peculiarity of non-ideal vapor flows in ORCs, measurements such as velocity magnitude and direction, mass flow rate and turbine performance, which are routinely carried out in more standard cycles and turbomachinery (e.g., gas turbines operating with air and combustion gases), are not often performed yet. This is mainly because no appropriately calibrated instrumentation for non-ideal conditions is currently available. Indeed, none of the previously mentioned wind tunnels for non-ideal flows is routinely employed as a dedicated calibration facility for pressure probes.

Research efforts are now starting to move toward this direction. Results on the performance of a rotatable cylinder Pitot probe in high-subsonic flows with fluid $\hbox {Novec}^{\text {TM}}$ 649 at the CLOWT plant were very recently presented (Reinker et al. 2020) as part of a preliminary study in order to establish measurement techniques for determination of Mach numbers in high-subsonic and transonic organic vapor flow fields.

The present authors also developed a pneumatic system for pressure probe measurements in flows of organic vapors in non-ideal conditions that sets the foundation for future directional pressure probes calibration and use in the characterization of such flows (Conti et al. 2022), and which is indeed the basis of the work here documented.

Considering the above, even blade cascade testing, quite common in the design process of gas and steam turbines, is instead significantly more complex in the case of non-ideal flows due to the previously mentioned difficulties in running dedicated wind tunnels and the lack of established probe measurement methodologies. Cascade testing is recently starting to take place for such flows. To the authors’ knowledge, the first experimental campaign of this kind was carried out at Whittle Laboratories of Cambridge University in a newly modified transient wind tunnel of Ludwieg tube type, where annular turbine cascade flows of R134a were characterized with pressure measurements (Baumgärtner et al. 2019). Wake measurements of R134a flows in the same cascade were taken with a wedge total pressure probe with substantial complementary use of CFD calculation. The latter was necessary to overcome the unavailability of a Mach number measurement upstream of the probe allowing to calculate shock losses at the probe tip in order to retrieve the pre-shock total pressure and evaluate cascade losses (Baumgärtner et al. 2020).

Linear blade cascade testing was recently carried out in the CLOWT facility at Muenster University in order to verify theoretical predictions of critical and choking Mach numbers of organic vapor flows (aus der Wiesche et al. 2021).

With the objective of measuring cascade losses to validate low- and high-fidelity turbine design tools, a linear cascade experiment representative of the stator operation of axial/radial turboexpanders for ORC systems will be concluded in the very near future at the TROVA wind tunnel at CREA Lab of Politecnico di Milano with siloxane MM (Manfredi et al. 2021).

Given the context above, the objective of the present research is twofold. A probe is inserted in non-ideal supersonic flow of siloxane MM (commonly employed in medium-/high-temperature ORCs) causing a detached bow shock locally normal to its tip. The first tests of this kind are here carried out to verify the correct functioning of the complete measurement system for direct shock loss measurements, with a view of establishing reliable experimental procedures. These will be fundamental for accurate measurements in non-ideal flows of particular interest for organic Rankine cycle applications, such as in blade cascades testing (Manfredi et al. 2021).

The experimental campaigns here reported also allow to directly characterize non-ideal shock losses at varying levels of non-ideality in operating conditions representative of the first-stage stator of ORC turbines, thus contributing to filling the current literature gap in available experimental data.

The present paper is structured as follows.

Section 2 illustrates the facility, instrumentation, nozzle and pneumatic system employed in the experimental campaigns here considered.

Section 3 reports results on preliminary tests with nitrogen as operating fluid.

Section 4 analyzes experimental campaigns with siloxane MM, with particular emphasis on highlighting the effects of flow non-ideality.

Section 5 draws the conclusions of this work and suggests future outlooks.

## Experimental setup

### Test Rig for Organic VApors - TROVA

The *Test Rig for Organic VApors* (TROVA) is a blowdown wind tunnel built with the aim of characterizing non-ideal flows of organic vapors, especially those representative of turbine expansions in ORCs. Different fluids can be employed, and experiments reported here were carried out with fluid siloxane MM (hexamethyldisiloxane, $\hbox {C}_6$ $\hbox {H}_{18}$ $\hbox {OSi}_2$), commonly employed in medium-/high-temperature ORCs. The working fluid is isochorically heated in a high-pressure vessel (HPV) until desired temperature and pressure are reached. It is then discharged to a low-pressure vessel (LPV) by passing through a settling chamber (plenum) and expanding in the test section where planar nozzles or blade cascades are located. Before the beginning of a test, the test section and LPV are vacuumized to MM saturation pressure at room temperature ($P\sim 50~\hbox {mbar}$). After test start, a peak is reached, and then, pressure in the test section decreases in time with a low-frequency content ($\sim 1~\hbox {Hz}$) related to the emptying of the HPV (Spinelli et al. 2018). Due to the decreasing pressure linked to the plant batch nature, the most non-ideal flow conditions are achieved at the beginning of each test. A more detailed description of the plant and its design can be found in (Spinelli et al. 2013).

### Nozzle expansion characterization and instrumentation

Flow expansions are characterized by total conditions measurements in the plenum upstream of the test section, where velocity is low enough (about ${1}{\text {m/s}}$) for kinetic energy to be negligible. Thus, total pressure $P_\text {T}$ is measured at a wall tap with an absolute pressure transducer and total temperature $T_\text {T}$ with thermocouples. In the present experimental campaigns with fluid MM, total temperature and pressure vary in the range $215-230~^\circ \hbox {C}$ and $2-12~\text {bar}$, respectively, with a compressibility factor evaluated at total conditions between $Z_\text {T}=0.68-0.98$.

Flow along the nozzle axis is characterized by static pressure measurements performed by absolute transducers mounted on the test section rear plate, connected through a 30 mm-long line cavity system to machined wall taps of $0.3~\hbox {mm}$ in diameter. The frequency response of this line cavity system was estimated well above that required to capture the pressure level changes linked to the emptying of the HPV (Spinelli et al. 2018).

Back plate-mounted absolute piezoresistive transducers (*Kulite* sensors of XTEH series) are exposed to high-temperature organic vapor flows. Thus, due to their temperature sensitivity, transducers were calibrated both in pressure and temperature from vacuum to full scale ($3.5~\hbox {bar} \le FS \le 40~\hbox {bar}$) in the range $25-300~^\circ \hbox {C}$. Thermocouples (of type K and type J by *TERSID*) are calibrated in the same temperature range.

The expanded uncertainty is 0.07 % of the full scale for pressure sensors and about $1~^\circ \hbox {C}$ for thermocouples. Data sampling frequency for temperature and pressure measurements is $1~\hbox {kHz}$, and signals are averaged over 100 time instants yielding a time resolution of $0.1~\hbox {s}$.

Discrete pressure measurements are supported by the schlieren technique for the visualization of key flow features such as Mach lines, shock waves and expansion fans. The optical bench employed for TROVA tests exploits the front optical access and the mirror-polished back plate for a double-pass configuration, with images directly formed onto the sensor of a high-speed CMOS camera. The position of the knife is such that positive density gradients (compressions and shock waves) appear dark while negative density gradients (expansions and expansion fans) appear bright. Further details on all TROVA instrumentation can be found in (Spinelli et al. 2018).

**Fig. 1:**
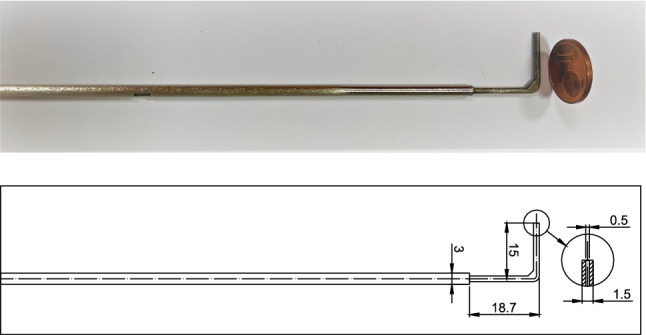
Total pressure probe employed for direct shock loss measurements. Dimensions are in mm

The total pressure probe in Fig. 1, with a stem length of $15~\hbox {mm}$, outer diameter of $1.5~\hbox {mm}$ and total pressure tap diameter of $0.5~\hbox {mm}$, is employed to perform direct shock loss measurements.

### Nozzle geometry

**Fig. 2:**
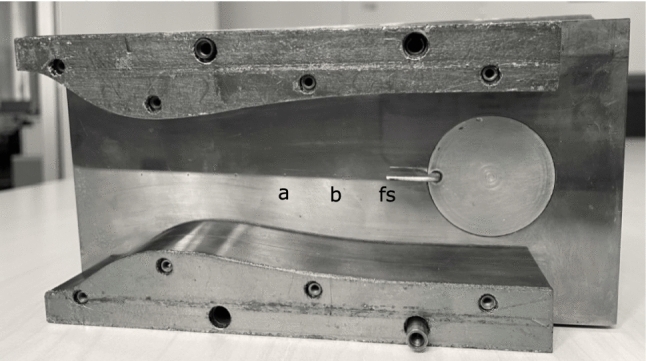
Test section back plate with labeled pressure taps (*a*, *b* and free-stream *fs*), with mounted nozzle profiles and pressure probe. The free-stream tap is hidden from view by the probe tip

**Fig. 3:**
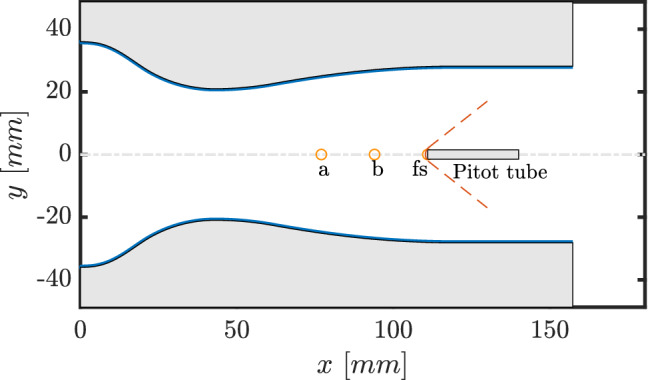
Nozzle with pressure taps (*a*, *b* and free-stream *fs*) and Pitot tube with a bow shock sketch

The experimental campaign was carried out employing the nozzle illustrated in Figs. 2 and 3, with the convergent section designed using a fifth-order polynomial, yielding a double concavity that provides gentle flow acceleration up to the throat and reduces flow disuniformities. The diverging portion shape is determined through the *method of characteristics* (MOC), implemented according to Zucrow and Hoffman (1977) and coupled with a suitable thermodynamic model for non-ideal gases Guardone et al. (2013) to deliver a uniform Mach number and a velocity parallel to the nozzle axis at the exit section. Further design details can be found in Zocca et al. (2019). The geometry yields a Mach number $M\sim 1.5$ with fluid siloxane MM at the free-stream pressure tap, indicated with *fs* in Figs. 2 and 3. The probe tip is placed exactly in correspondence of this tap to provide a precise pre-shock static pressure reference. Although the distance between the probe head axis, located at the channel center, and the back plate is relatively small ($\sim 9.3~\hbox {mm}$), no shock boundary layer interaction was found to occur at the position of the free-stream wall tap. This was assessed through comparison of tests in this same nozzle both with and without probe insertion (not reported here for brevity), which revealed a free-stream pressure unaltered by the presence of the probe and the related shock wave.

As reported in Fig. 3, nozzle length is $157.3~\hbox {mm}$, with semi-heights of $36.0~\hbox {mm}$ at the inlet, $21.0~\hbox {mm}$ at the throat and $28.2~\hbox {mm}$ at the outlet. Depth (distance in the nozzle spanwise direction) is imposed by the test section to $18.7~\hbox {mm}$.

### Nitrogen-flushed pneumatic system

**Fig. 4:**
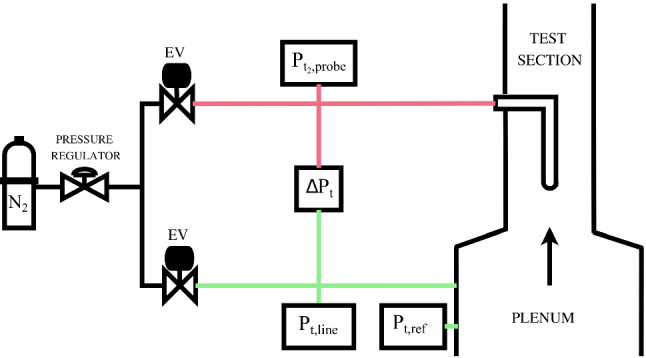
Pneumatic lines scheme in the TROVA for shock total pressure loss measurement with probes. Rectangular boxes represent pressure transducers

When probes are employed, pneumatic lines are inevitably present. Since siloxane MM is liquid at room temperature and considered operating pressures during tests, unheated pneumatic lines are subject to condensation, leading to poor measurement quality related to the presence of vapor–liquid menisci, hydrostatic head and time delay due to mass sink effects. The pneumatic system documented in Conti et al. (2022) involving nitrogen flushing was implemented to allow probes insertion in the TROVA test section while avoiding the above issues. The configuration was experimentally optimized to minimize the overall volume (including tubing, fittings and transducers volumes) to ensure a response time that is appropriate for resolving the blowdown dynamics of the plant. Figure 4 illustrates the configuration employed in the present experimental campaigns, essentially composed by two lines: one exiting the plenum for pre-shock total pressure measurement and one connected to the probe tap measuring post-shock total pressure. Both lines are directly connected to a nitrogen storage tank. Electrovalves are actuated to open as the test is triggered to flush lines with nitrogen at a pressure just above the maximum expected one during the test, and close right after the pressure peak is reached in the test section. This ensures that each line only contains nitrogen at all times during a test and no MM vapor enters it, so as to avoid condensation. As the test proceeds, nitrogen exits the line through the static tap into the test section as line pressure is in equilibrium with the decreasing test section one.

Shock total pressure loss is directly measured as $\Delta P_\text {t}$, the difference between plenum total pressure (upstream of the shock) and probe total pressure (downstream of the shock). A differential transducer was employed to minimize measurement uncertainty, specifically, a *Kulite* sensor of *XTL-3-375 (M)* series with $5.9~\hbox {bar}$ full scale and an expanded uncertainty of $10~\hbox {mbar}$. Absolute transducers (of the same type as plate-mounted ones for nozzle axis measurements) are present on each line to help identify possible issues: $P_{\text {t,line}}$ measures the pre-shock total pressure in the line exiting the plenum and $P_{\text {t2,probe}}$ measures total pressure in the line connected to the probe. A pre-shock total pressure reference measurement $P_{\text {t,ref}}$ is also carried out in the TROVA plenum with a wall-mounted absolute transducer.

## Shock loss measurements in supersonic nitrogen flow

**Fig. 5:**
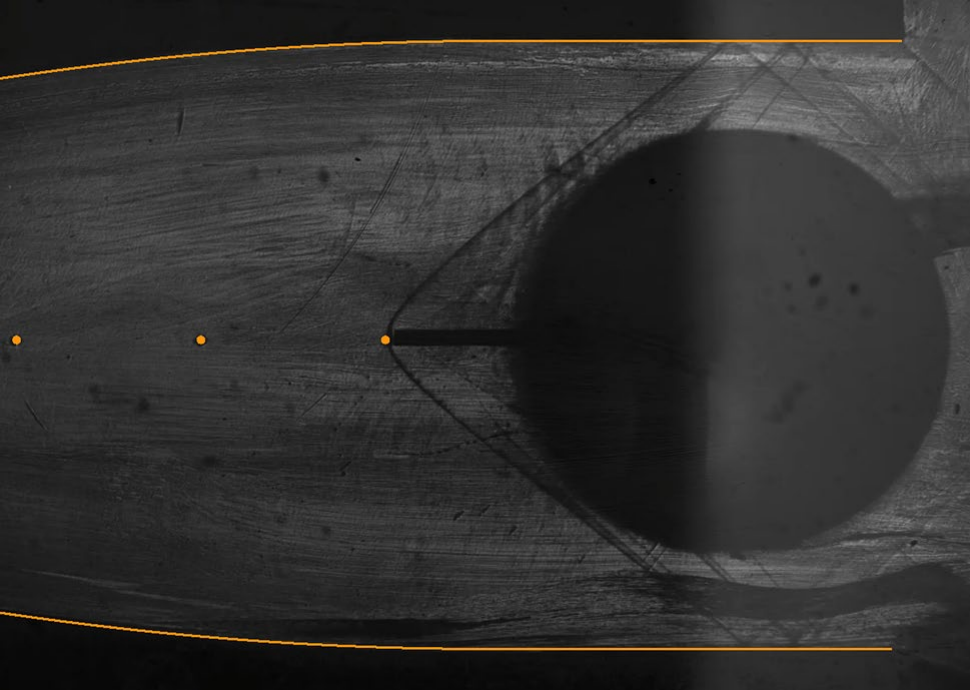
Schlieren image at $t=7~\hbox {s}$ during preliminary test TROVA-N$_2$. Nozzle contour and pressure taps are highlighted. The dark circle is the shaft employed to insert the probe in the test section and shows little light reflection due to poor local quality of the surface finish

Preliminary tests with nitrogen were carried out prior to the experimental campaign with MM in order to assess the measurement system performance. Reliability of experimental results was verified through repeated testing, although only one exemplary test (TROVA-N$_2$) is reported here for brevity. The schlieren image in Fig. 5 shows that the wind tunnel operating regime is indeed the expected one, with supersonic flow impinging on the probe tip resulting in a bow shock that is locally normal to it.

**Fig. 6:**
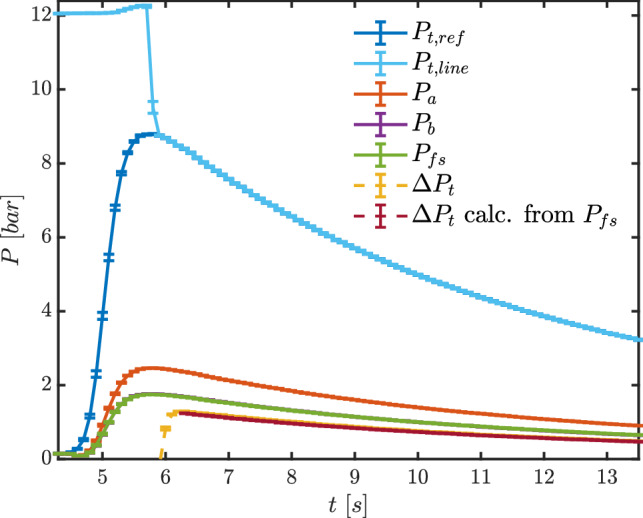
Test TROVA-N$_2$: pressures during test time. Line flushing is present until $t=5.7~\hbox {s}$ for $P_{\text {t,line}}$ and $\Delta P_\text {t}$

Figure 6 illustrates measured pressures during test TROVA-N$_2$. It was performed with line flushing to test the full procedure for MM experimental runs, although it was unnecessary here due to the impossibility of condensation. Pressure $P_{\text {t,line}}$ is in perfect agreement with the reference total pressure in the plenum $P_{\text {t,ref}}$ after line flushing has ended, indicating no issues on the upstream total pressure line to the differential transducer. The two pressures are within error bars of one another from test time $t=5.9~\hbox {s}$, so less than $0.2~\hbox {s}$ after nitrogen flushing was stopped at pressure peak. Flushing pressure was chosen as representative of expected peak pressure with MM and is slightly high for the present nitrogen testing conditions.

Pressure $P_\text {a}$ is larger with respect to $P_\text {b}$ and $P_{\text {fs}}$, consistently with tap position along the nozzle, as per Figs. 2 and  3.

The plot also reports the total pressure loss across the shock $\Delta P_\text {t}$ directly measured with the differential transducer. It is compared to the theoretical shock loss calculated from total conditions, measured $P_{\text {fs}}$ and assuming a normal shock at the probe tip. $\Delta P_{\text {t}}$ *calc. from* $P_{\text {fs}}$ was determined by numerically solving mass, momentum and energy conservation equations across the shock coupled with the Span–Wagner thermodynamic model through the *FluidProp* library (Span and Wagner 2003). This is a state-of-the-art multiparameter model that provides accurate thermodynamic properties even close to the critical point. A functional form in terms of the reduced Helmoltz free energy, as a function of the inverse reduced temperature and reduced density, is provided for the fundamental relation linking all thermodynamic properties of a simple system in a stable equilibrium state. For siloxane MM, implemented model parameters were first reported in Colonna et al. (2006, 2008) and were more recently improved by Thol et al. (2016, 2017).

Analytical Rankine–Hugoniot equations for a polytropic ideal gas with specific heat ratio $\gamma =1.4$ were used to verify the above shock loss calculation method, which is generalized to non-ideal flows, for following use with MM.

The uncertainty associated with calculated shock total pressure loss was determined by propagating measurement uncertainty in total temperature, total pressure and free-stream pressure. Given the very large number of calls to the thermodynamic model, Monte Carlo-like methods are too computationally expensive, and a procedure based on polynomial chaos expansions was thus implemented using the *UqLab* framework (Marelli and Sudret 2021) .

**Fig. 7:**
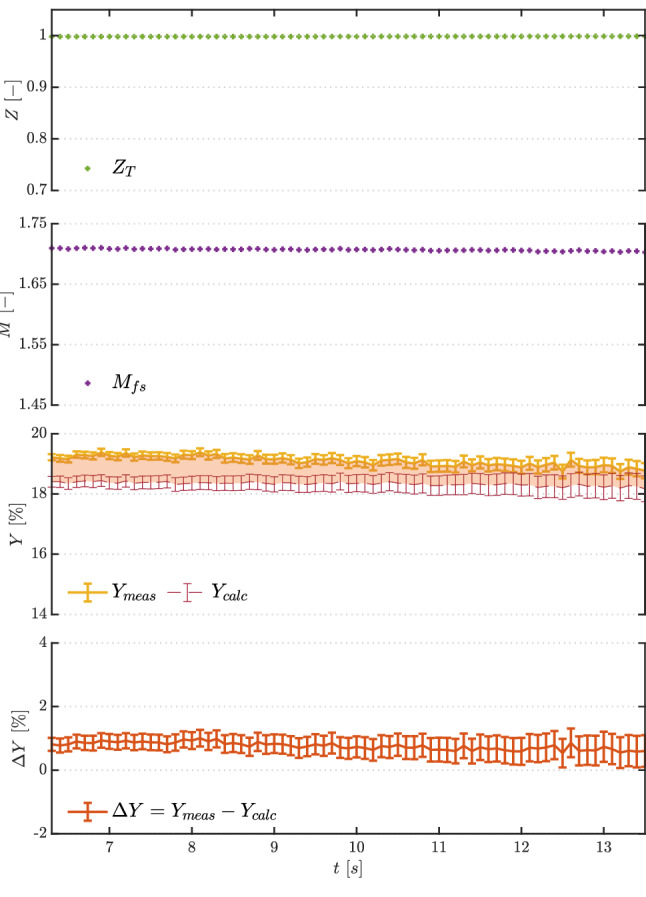
Test TROVA-N$_2$: zoom at time $t=6.3-13.5~\hbox {s}$ for compressibility factor at total conditions $Z_\text {T}$, free-stream Mach number $M_{\text {fs}}$, measured $Y_{\text {meas}}$ and calculated $Y_{\text {calc}}$ loss coefficients, and their difference $\Delta Y$

The top two plots in Fig. 7 illustrate trends in the compressibility factor evaluated at total conditions $Z_\text {T}$ and in the free-stream Mach number $M_{\text {fs}}$. The latter is calculated from total pressure, total temperature and measured free-stream pressure using the Span–Wagner thermodynamic model and assuming an isentropic flow. Both quantities confirm the polytropic ideal gas behavior of the flow, with constant values of $Z_\text {T}=1$ and of $M_{\text {fs}}\simeq 1.7$ that are independent of the actual total conditions.

To evaluate loss measurements quality, the loss coefficient *Y* was defined as:

2 $$\begin{aligned} Y = \frac{\Delta P_{\text {t,shock}}}{P_{\text {t,ref}}-P_{\text {fs}}} \end{aligned}$$

where the numerator represents shock total pressure loss and the denominator is pre-shock kinetic head. The loss coefficient was calculated using both the measured total pressure difference and the one calculated from $P_{\text {fs}}$, as reported in the third plot from the top in Fig. 7, and referred to in the legend as $Y_{\text {meas}}$ and $Y_{\text {calc}}$, respectively. Both losses show a constant value during the test, highlighting the ideal gas behavior of nitrogen at the considered mild thermodynamic conditions, given the independence of shock losses from the varying total quantities.

The difference $\Delta Y = Y_{\text {meas}} - Y_{\text {calc}}$ (bottom plot in Fig. 7) is also fairly constant and below $1\%$, with the agreement between measured and theoretical shock losses indicating a good measurement system performance.

## Shock loss measurements in supersonic siloxane MM flow

**Fig. 8:**
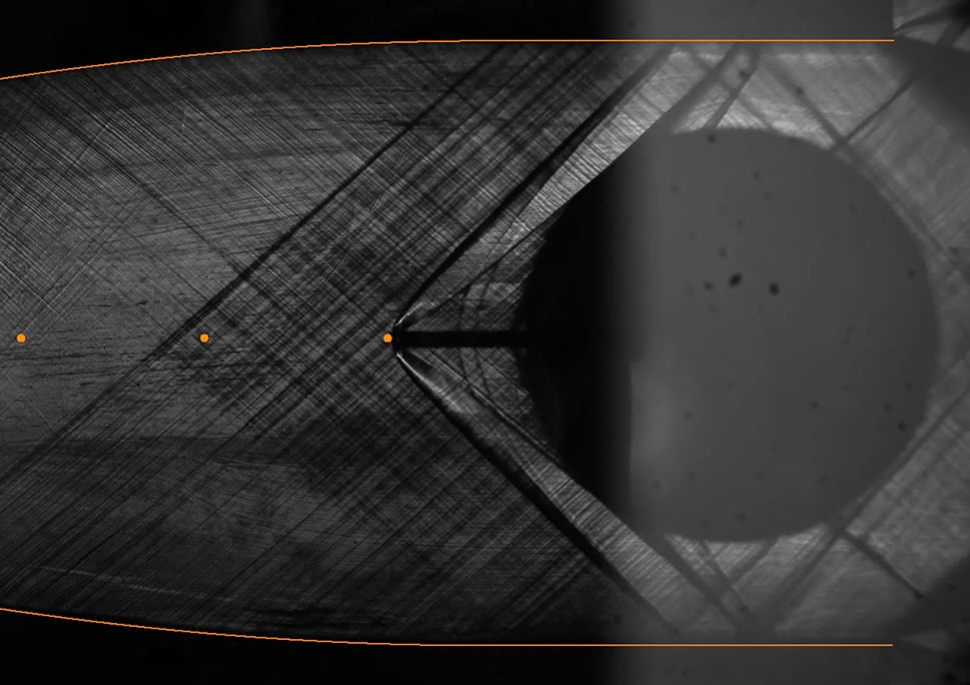
Schlieren image at $t=5.2~\hbox {s}$, corresponding to $Z_\text {T}=0.70$ during test TROVA-MM. Nozzle profile and pressure taps are highlighted

The schlieren image in Fig. 8 shows that the wind tunnel operates as intended during experimental campaigns with siloxane MM too. Analogously to test TROVA-N$_2$ with nitrogen, supersonic flow impinges on the probe tip with a bow shock that is locally normal to it.

**Fig. 9:**
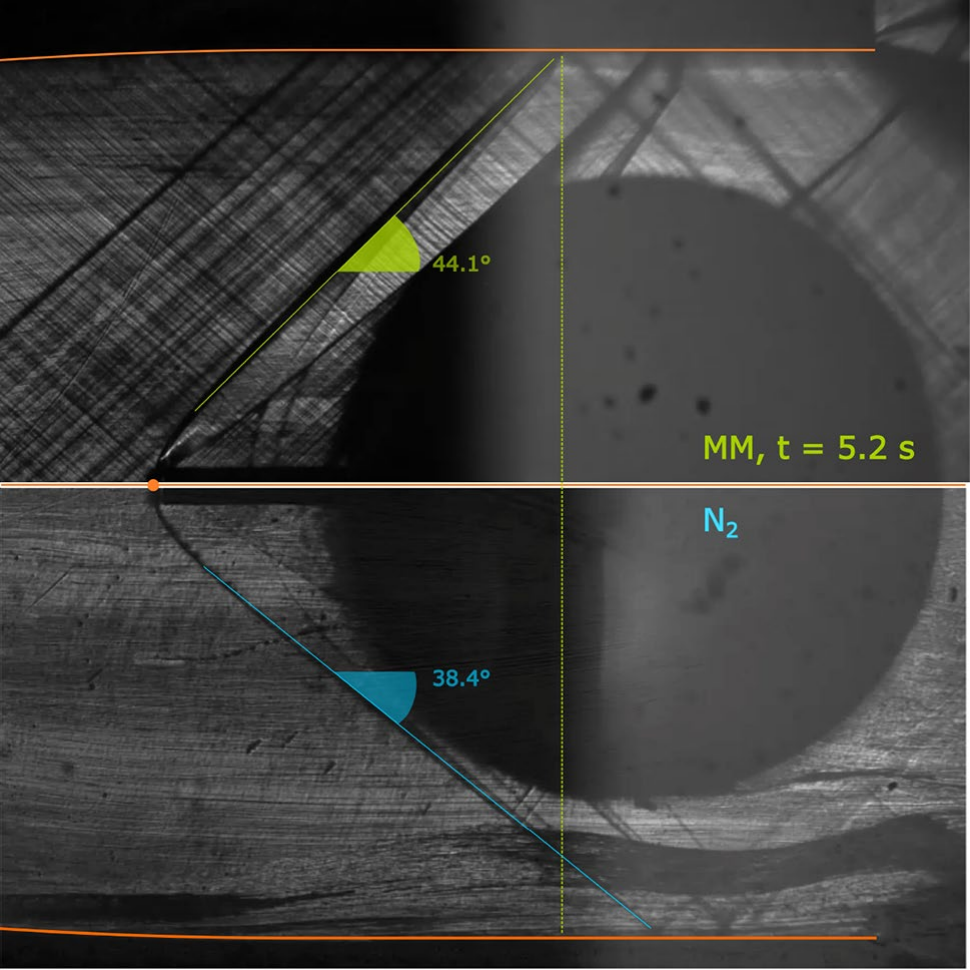
Zoom-in on two schlieren images during test TROVA-MM at $t=5.2~\hbox {s}$ above and test TROVA-N$_2$ below

As evidenced in Fig. 9, the higher shock slope compared to nitrogen indicates a lower pre-shock Mach number, linked to the higher siloxane MM molecular complexity. Indeed, since nozzle geometry is fixed, nitrogen flow reaches a pre-shock Mach number of $M_{\text {fs}}\simeq 1.7$, while MM vapor is in the range $M_{\text {fs}}=1.47-1.57$, depending on total conditions.

The stronger density gradients compared to test TROVA-N$_2$ allow to visualize the Mach lines due to surface roughness of nozzle profiles.

**Fig. 10:**
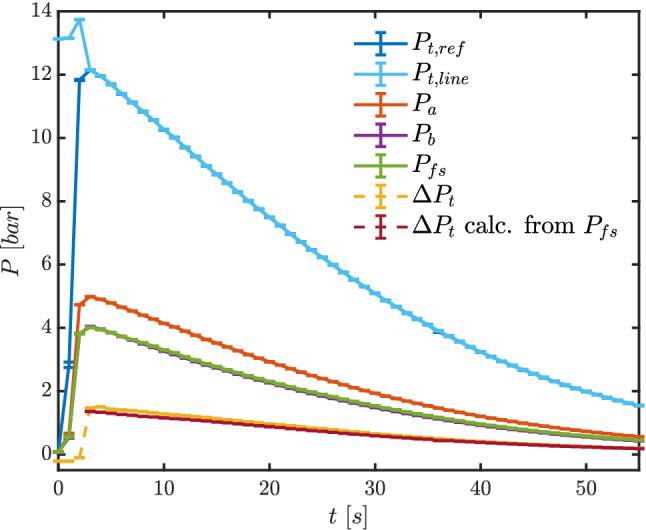
Test TROVA-MM: pressures during test time. Line flushing is present until $t=2.5~\hbox {s}$ for $P_{\text {t,line}}$ and $\Delta P_\text {t}$

Reliability of experimental results was verified through repeated testing (not reported for brevity), and only exemplary test TROVA-MM is analyzed here, with measured pressures reported in Fig. 10. Pressures $P_{\text {t,line}}$ and $P_{\text {t,ref}}$ are always within error bars of one another throughout the test after line flushing has ended. Pressure $P_\text {a}$ is larger with respect to $P_\text {b}$ and $P_{\text {fs}}$, consistently with tap position along the nozzle. Figure 10 also shows the total pressure loss across the shock $\Delta P_\text {t}$ directly measured with the differential transducer, and the one calculated from total conditions, $P_{\text {fs}}$ and balance equations across the shock (together with its associated propagated uncertainty), as previously explained.

**Fig. 11:**
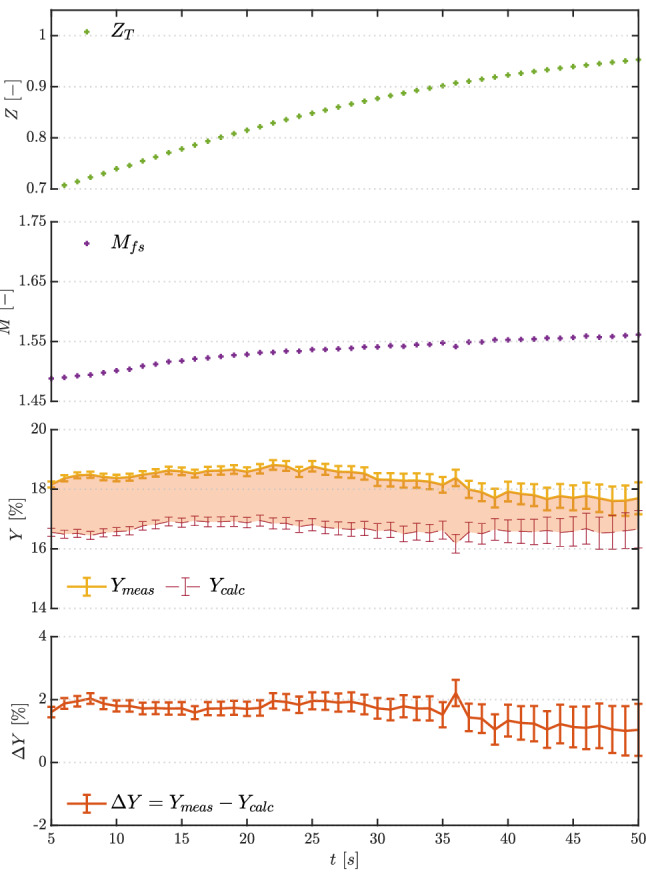
Test TROVA-MM: zoom at time $t=5-50~\hbox {s}$ for compressibility factor at total conditions $Z_\text {T}$, free-stream Mach number $M_{\text {fs}}$, measured $Y_{\text {meas}}$ and calculated $Y_{\text {calc}}$ loss coefficients, and their difference $\Delta Y$

The two top plots in Fig. 11 report trends in the compressibility factor at total conditions $Z_\text {T}$ and in the free-stream Mach number $M_{\text {fs}}$. Contrarily to nitrogen, both quantities vary significantly. $Z_\text {T}$ increases from values just below 0.7 toward 1 during the test, indicating more dilute conditions as the test proceeds. The free-stream Mach number has a value of $M_{\text {fs}}=1.47$ at test start and increases to $M_{\text {fs}}=1.57$, showing a marked non-ideal dependence on total pressure and temperature. The loss coefficient *Y*, defined in Eq. 2, is also reported in Fig. 11 for measured and calculated losses, together with their difference $\Delta Y$. Contrarily to test TROVA-N$_2$ with nitrogen, losses are not constant during testing with siloxane MM, again highlighting the non-ideal dependence of the flow field on total conditions. The agreement between experimental and theoretical loss coefficient is satisfactory, with a difference always below $2 \%$ throughout the test, with a slight decrease toward more ideal conditions to values consistent with nitrogen testing.

### Evidence of flow non-ideality across shocks

**Fig. 12:**
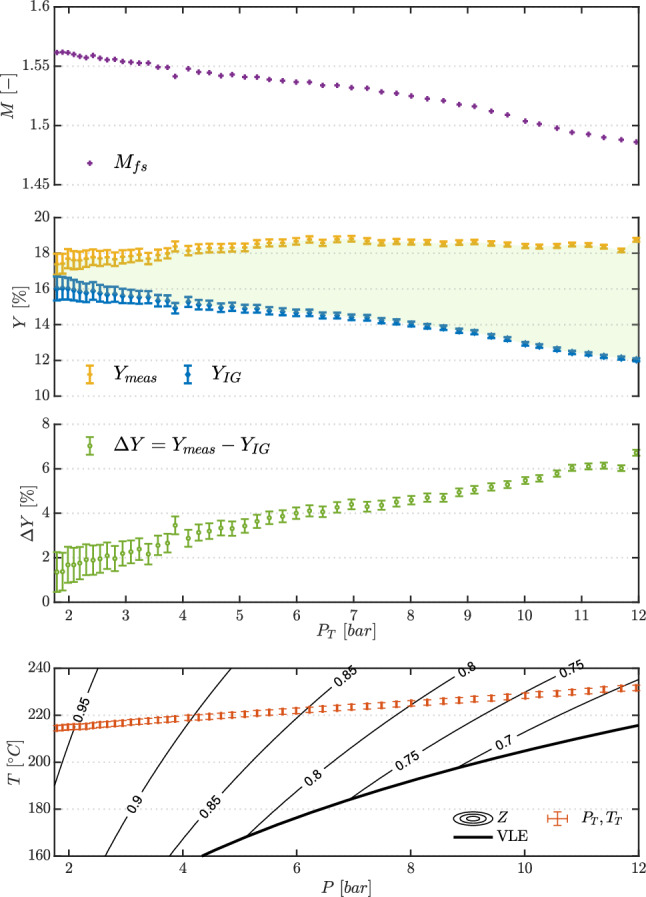
Quantities of interest during test TROVA-MM. From the top: free-stream Mach number (error bars omitted), measured and calculated losses, their difference, and evolution of total pressure and temperature in the *T–P* plane with compressibility factor *Z* contours and vapor–liquid equilibrium (VLE) curve

Figure 12 allows to analyze non-ideality effects with quantities plotted in terms of total pressure, which substitutes the test time reference. Initial time instants correspond to higher total temperature and pressures, and to lower values of compressibility factor evaluated at total conditions $Z_\text {T}$, as represented in the *T*–*P* plot at the bottom of the figure.

The blowdown nature of the plant means that total conditions change as the test proceeds. Given non-ideality, this leads to a varying free-stream Mach number, with higher values toward the end of the test at lower pressure levels, as previously pointed out. Values of free-stream Mach number as a function of total pressure are reported in Fig. 12, at the top. At higher pressure levels at test start, $M_{\text {fs}}=1.47$, increasing to $M_{\text {fs}}=1.57$ at lower pressures.

**Fig. 13:**
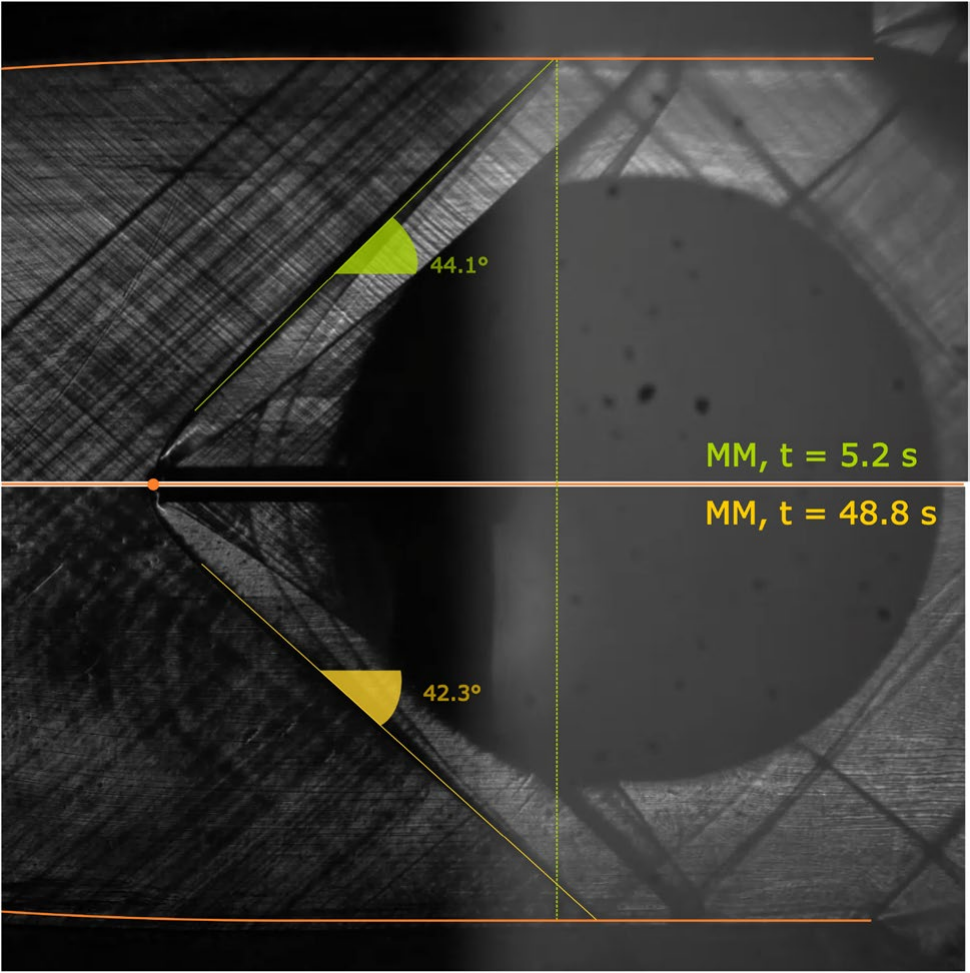
Zoom-in on two schlieren images during test TROVA-MM: $t=5.2~\hbox {s}$ at $Z_\text {T}=0.70$ above, $t=48.8~\hbox {s}$ at $Z_\text {T}=0.95$ below

Qualitatively, this is confirmed by Fig. 13, where frames corresponding to two different time instants of the schlieren video are compared. The top image is at test start, with significant non-ideality and a compressibility factor evaluated at total conditions of $Z_\text {T}=0.70$, while the bottom one is close to ideal gas behavior with $Z_\text {T}=0.95$. The shock wave is less steep at more ideal conditions, indeed indicating a higher free-stream Mach number.

Therefore, due to the specific TROVA operating mode for a single test run, the pre-shock Mach number cannot be kept constant at different levels of non-ideality. Thus, to highlight and isolate the impact of the latter, ideal gas shock losses $Y_{\text {IG}}$ are reported in the second plot from the top in Fig. 12. These are computed from $M_{\text {fs}}$ by employing the value of shock total pressure losses from analytical Rankine–Hugoniot equations for a polytropic ideal gas. The employed value of the specific heat ratio $\gamma$ is calculated in the ideal gas limit, that is, at free-stream temperature $T_{\text {fs}}$ (computed analogously to $M_{\text {fs}}$) and at pressure $P \rightarrow 0$.

In this way, the difference between $Y_{\text {IG}}$ and the measured $Y_{\text {meas}}$ is entirely due to non-ideality, which directly impacts shock intensity leading to higher losses for a given free-stream Mach number. As expected, the effect is more marked in more non-ideal conditions at higher pressures, with a difference compared to the ideal gas limit reaching $\Delta Y=6\%$.

Incidentally, during a single run at the total temperatures and pressures tested in the present experimental campaign, the variation of plant operating conditions and direct effects of non-ideality on shock intensity in fact compensate each other. At higher pressure levels, the free-stream Mach number is lower, which should bring to lower losses if the behavior were that of an ideal gas across the shock. However, this is offset by the stronger shock intensity linked to the more non-ideal conditions, an effect which fades as dilute gas is approached. The overall result is a value of $Y_{\text {meas}}$ which increases and then decreases slightly as the test proceeds, but is settled around the same value.

It is nevertheless remarkable to verify that non-ideality effects have a direct significant impact on shock intensity, even at the mildly non-ideal conditions with $Z_\text {T}\gtrsim 0.70$ considered here.

The ideal gas model assumes identical point particles interacting with each other only through elastic collisions, and not subject to forces of mutual attraction or repulsion. Generally, a gas behaves as an ideal gas at high temperatures and low pressure, far away from the vapor–liquid equilibrium curve, because the potential energy related to intermolecular interactions is negligible with respect to kinetic energy, and molecules’ volume is negligible compared to the surrounding empty space. In case of complex fluids with high molecular mass, as pressure increases and conditions are close to vapor–liquid equilibrium, co-volume and intermolecular interaction forces are extremely relevant, causing a significant departure from the ideal gas behavior. In the present case of siloxane MM at the considered operating conditions, this results in larger total pressure shock losses compared to polytropic ideal gas behavior at same pre-shock conditions. Analogous trends were observed in a theoretical analysis reported in Passmann et al. (2017) for fluid $\hbox {Novec}^{\text {TM}}$ 649.

## Conclusions and outlook

The present work documents the first ever direct measurements of total pressure losses across shocks in non-ideal supersonic flows representative of first-stage turbine stators in organic Rankine cycles.

A pneumatic system involving nitrogen line flushing was implemented to allow pressure probe measurements in the test section of the *Test Rig for Organic VApors* blowdown wind tunnel at Politecnico di Milano.

Preliminary tests with nitrogen at free-stream Mach number $M_{\text {fs}}\simeq 1.7$ were carried out to verify the measurement procedure in ideal gas flows.

Experimental campaigns were then carried out with siloxane MM vapor at free-stream Mach number $M_{\text {fs}} \sim 1.5$, with varying total conditions and level of non-ideality. Direct total pressure measurements were compared with losses calculated using pre-shock quantities and by solving conservation equations across a normal shock employing a state-of-the-art thermodynamic model. Observed differences were always below $2 \%$, attesting the satisfactory reliability of the implemented experimental procedure.

Test results were then analyzed by separating the impact of pre-shock Mach number variation due to the blowdown nature of the plant, and direct non-ideality effects on shock intensity. As a general finding, the campaign here reported allowed to verify that non-ideality has a direct significant impact on shock intensity. Even at the considered mildly non-ideal conditions with $Z_\text {T}\gtrsim 0.70$, it was responsible for a stronger shock compared to the ideal gas limit at same free-stream Mach number, with differences as large as $6\%$.

The present research contributes to establishing reliable experimental procedures for shock loss measurements in non-ideal flows. This sets the foundations for future pressure probes calibration and use for characterization of non-ideal supersonic flows of organic vapors, such as in the testing of ORC turbine blade cascades. Moreover, the results here reported contribute to filling the literature gap concerning experimental data on shock losses in non-ideal flows and are thus useful for the validation of related numerical design and analysis tools.
